# Determination of the minimum number of microarray experiments for discovery of gene expression patterns

**DOI:** 10.1186/1471-2105-7-S4-S13

**Published:** 2006-12-12

**Authors:** Fang-Xiang Wu, WJ Zhang, Anthony J Kusalik

**Affiliations:** 1 Department of Mechanical Engineering, University of Saskatchewan, Saskatoon, SK, S7N 5A9, Canada; 2 Division of Biomedical Engineering, University of Saskatchewan, Saskatoon, SK, S7N 5A9, Canada; 3 Department of Computer Science, University of Saskatchewan, Saskatoon, SK, S7N 5C9, Canada

## Abstract

**Background:**

One type of DNA microarray experiment is discovery of gene expression patterns for a cell line undergoing a biological process over a series of time points. Two important issues with such an experiment are the number of time points, and the interval between them. In the absence of biological knowledge regarding appropriate values, it is natural to question whether the behaviour of progressively generated data may by itself determine a threshold beyond which further microarray experiments do not contribute to pattern discovery. Additionally, such a threshold implies a minimum number of microarray experiments, which is important given the cost of these experiments.

**Results:**

We have developed a method for determining the minimum number of microarray experiments (i.e. time points) for temporal gene expression, assuming that the span between time points is given and the hierarchical clustering technique is used for gene expression pattern discovery. The key idea is a similarity measure for two clusterings which is expressed as a function of the data for progressive time points. While the experiments are underway, this function is evaluated. When the function reaches its maximum, it indicates the set of experiments reach a saturated state. Therefore, further experiments do not contribute to the discrimination of patterns.

**Conclusion:**

The method has been verified with two previously published gene expression datasets. For both experiments, the number of time points determined with our method is less than in the published experiments. It is noted that the overall approach is applicable to other clustering techniques.

## Background

Recent advances in microarray technologies [1,2] and genome sequencing have allowed the expression level of thousands of genes to be monitored in parallel. This tool provides important new information for the fundamental understanding of biological processes at the molecular level. Such understanding has proved very useful in medical diagnosis, disease treatment, and drug design. From a viewpoint of data analysis, microarray experiments may be categorized into (1) classification of subjects into various subtypes based on gene expressions, (2) discovery of gene expression patterns over a set of conditions, and (3) discovery of gene expression patterns for a cell line over a series of time points while a biological process is underway. This paper concerns the third category of experiments, which may also be called temporal gene expression pattern discovery. The biological significance of understanding temporal gene expression patterns has been well recognized; see Eisen et al. [3].

An important feature of this category of microarray experiments is dependency among gene expression data corresponding to different time points. An important issue is thus the specification of time points, including their number and the span between them. In the absence of knowledge from the biologist about this specification, one naturally questions whether the behaviour of data generated from a progressive microarray experiment may help by itself determine a "cut off", beyond which further micorarray experiments do not contribute to the discrimination of gene expression patterns. Additionally, such a cut-off value implies the minimum number of microarray experiments, which is important because these experiments are costly in terms of time, reagents, and well-trained technicians [4,5]. Therefore, it is useful to develop a method for determining the minimum number of time points required to obtain useful patterns for this class of microarray experiments. The study reported in this paper develops such a method. Initially we assume that the interval between consecutive time points is given (preset). Later in the document, this assumption is discussed further.

A clustering technique is typically used for discovering patterns in gene expression data. There are many clustering algorithms available in literature for gene expression profiling, including the hierarchical clustering [6], K-means clustering [7], self-organized maps [8] and mixture model-based clustering [9]. In this study, we adopt hierarchical clustering technique. However, the method for determination of the minimum number of microarray experiments can be similarly developed with other clustering techniques.

There appears to be only few related studies in the literature. The most related work may refer to Hwang et al. [5], in which a method was developed for determining the minimum sample size in the context of supervised classification of gene expression data. Their method has addresses the problem with the first category of experiments. In Hwang's study [5], they assumed that different samples were statistically independent, and thus they were able to apply power analysis in statistics. However, their method cannot be applied to the third category of experiments because temporal gene expression data are dependent to each other in the time parameter and thus are not statistically independent. Other related studies include those of Lee et al. [10] and Pan et al. [11]. Lee et al. [10] studied a generic problem of determining the number of replicates needed for producing high-quality gene expression data. Their method was based a mixed probability density function with two normal distributions. Pan et al. [11] further extended Lee et al.'s model into a mixture of a number of normal distributions.

There are two key ideas in this paper. First, a statistics-based similarity measure for two clusterings produced with the hierarchical clustering technique is defined. Second, a procedure is developed for determining whether an experiment after time point *k *further contributes to the identification of patterns. The procedure compares two clusterings based on data over the first *m *- 1 and *m *time points.

## Results and Discussions

To evaluate the proposed method, a program implementing it was run on two datasets: the fibroblast dataset and the cdc15 dataset (see the "Methods" section for the details about these datasets). The function *c*(*m*) is employed to measure the similarity of two clusterings based on expression data over the first *m *and *m *- 1 time points (see the "Method" section for the definition). Figures 2 and 3 depict the profiles of *c*(*m*) with respect to the number of time points *m *for the fibroblast dataset and for the cdc15 dataset, respectively. Correspondingly, Tables 2 and 3 list the numerical values of *c*(*m*). It can be seen from these two figures that the *c*(*m*) values in both datasets initially increase monotonically with respect to the number of time points, reach a maximum, and then appear to randomly fluctuate thereafter.

Table 2 and Figure 2 show that *c*(*m*) reaches an initial maximum when data from the first 9 time points are used to cluster genes and then appears to randomly fluctuate when more data are added. Therefore, it is reasonable to claim that nine is the minimum number of time points necessary for clustering genes for the fibroblast experiment. This result matches very well with the fact that the fibroblast dataset from the first nine time points were collected over the first 16 hours after serum stimulation, and the period of cell division is 16 hours (see Table 1). It should be noted that to detect the maximum, the tenth data sample needs to be added. Thus in fact, the whole experiment requires 10 time points.

Tables 3 and Figure 3 show that *c*(*m*) reaches an initial maximum when data from the first 8 time points are used to cluster genes and then appears to randomly fluctuate when more data are added. Again it is reasonable to claim that eight is the minimum number of time points necessary for clustering genes in the cdc15 experiment. This result also correlates very well with the fact that data from the first eight time points were collected over the first 100 minutes after cdc15-based synchronization, and the period of cell division is about 100 minutes (see Table 1). For the same reason as in the fibroblast case, the cdc15 experiment actually requires 9 time points.

The function *d*(*k*, *m*) is defined to measure the similarity of two *k*-partition clusterings based on expression data over the first *m *and *m *- 1 time points and obtained at a proper level of the corresponding hierarchical clusterings (see the "Method" section for the definition). In the following, we examine the behaviour of *d*(*k*, *m*) by setting *k *= 3, 4,⋯10, respectively. This is very important as we want to understand how the number of clusters, *k*, could affect the results, specifically, whether the minimum number of microarray experiments obtained with *c*(*m*) is applicable to different partitions. Figure 4 shows the profiles of *d*(*k*, *m*) for the fibroblast experiment for the various values of *k*. When the fourth sample is added, the probability that any two gene pairs are clustering-invariant for partition with 3 clusters is only about 60% while the probability for the partition with 10 clusters is about 80%. It is found that the possibilities for all possible partitions increase as more data are added. For instance, when the seventh sample is added *d*(3, *m*) reaches its initial maximum, and at this point, the probability that any two gene pairs are clustering-invariant is about 95%. When the eighth sample is added *d*(10, *m*) reaches a maximum for the first time, and at this point, the probability that any two gene pairs are clustering-invariant is about 94%. For other partitions, their corresponding values of *d*(*k*, *m*) reach their initial maxima before the ninth sample is added (see Figure 4).

It is interesting to observe from the above discussion regarding the behaviour of *d*(*k*, *m*) that fewer samples may be needed to obtain a *k*-partition when the number of clusters *k *is known as a priori information. This seems to be reasonable as more clusters require more discriminant features (i.e. more samples). However, there may exist a kind of 'saturated' *k *(i.e., the number of clusters), beyond which the increase of *k *will not call for more samples. For instance, the case of the fibroblast experiment, such a saturated number of clusters is 7, as the same number of samples (i.e., 8) is required for numbers of clusters more than 7. A similar situation can be observed for the cdc15 dataset.

## Conclusion

The method proposed in this paper to determine the minimum number of time points required in DNA microarray experiments for clustering genes has been shown to be effective by analyzing two previously published datasets: fibroblast and CDC15. These two datasets have temporal gene expression profiles with definite periods; specifically about 16 hours for the fibroblast and about 100 minutes for the cdc15. The periodic behaviours of these two datasets were observed in the originating experiments; specifically the number of time points is 12 for the fibroblast datasets, while the number of points is 24 for the cdc15 dataset. With our method, we obtain the following numbers of microarray experiments: 10 for the fibroblast experiment and 9 for the cdc15 experiment. These minima imply a significant reduction of time points (i.e., microarray experiments), especially for the cdc15 experiment.

Another finding is regarding the use of the average linkage method of the hierarchical clustering technique with Euclidean distance measure and the *γ*_*c *_measure for clustering similarity employed in our method. Overall our computational experiments have shown that such a combination appears to work well for applications, which is consistent with the result and conclusion obtained by Dougherty et al. [12]. Last, the index *D *is able to give detailed information about the object pair invariant property, which can be useful when the number of clusters is given perhaps by a biologist. In such a case, the number of microarray experiments can be further reduced.

There are several limitations with this study at its present stage. First, the span between two consecutive time points obviously affects the minimum number of microarray experiments required. The shorter the interval, the better the resolution of a time-series gene expression profile. The present study assumed that the interval is given. When the underlying biological processes do not suggest an appropriate interval, the study presented by Langmead et al. [13] is helpful. In it a computational approach was developed for determining a reasonable period between two consecutive time points. It is expected that the combination of their method and the method presented in this paper would further reduce the number of microarray experiments necessary for pattern discovery.

One of the problems with our method is that beyond the cut-off (i.e., the minimum number of experiments) both *c*(*m*) and *d*(*k*, *m*) functions fluctuate, which seems to challenge the legitimacy of our idea (i.e., to pick up the first maximum of *c*(*m*) or *d*(*k*, *m*)). Our experience is that such fluctuations should not be statistically significant. While a probabilistically sound proof of this statement is warranted, it would require a large number of samples, the cost of which is unaffordable by many labs.

Another problem lies in computational overhead with the agglomerative hierarchical clustering techniques. For an experiment with a large number of genes, the run time to perform a hierarchical clustering can be very long. There are two solutions to this problem. One is from a computation perspective, e.g., introducing a parallel algorithm or using a computer cluster. The other is to look into the biological process itself, screening for a small set of genes which play dominant roles. For example, Hwang et al. [5] first determined a set of dominant genes and then constructed a classifier with this reduced set of genes.

In conclusion, this study has produced a method for determining the minimum number of microarray experiments for collecting temporal gene expression data when the interval of time points is pre-specified from a biological viewpoint. The presented method works for the hierarchical clustering technique and for two situations: (1) the number of clusters is not given, and (2) the number of clusters is given. Although this method appears to be more useful for cyclic gene expression profiles (noticeably, the method has been validated by two sets of data from cyclic cell division processes), it can be used for any other situation. For example, it can be applied to microarray experiments monitoring the development or growth process of a cell.

## Methods

### Datasets and gene selection

Two previously published datasets from DNA microarray experiments were used in this study. Iyer et al. [14] studied the responses of human fibroblasts to serum and measured the temporal changes of 8613 human genes in mRNA levels at 12 time points, ranging from 15 minutes to 24 hours after serum stimulation. They selected 517 out of 8613 genes for their study. The selection criteria they used are: if either (i) their expression profiles have at least two log_2_-ratio values whose magnitudes are greater than log_2_(2.2); or (ii) their standard deviations for log_2_-transformed expression values exceeded 0.7. Our first dataset comes from Iyer et al.'s experiments and consists of expression data of these 517 genes at 12 time points. This dataset is available at .

Spellman et al. [15] studied the mitotic cell division cycle of yeast and monitored more than 6000 ORFs of yeast (*Saccharomyces cerevisiae*) at 24 time points in a cdc15-synchronized experiment. The original dataset is available at . Our second dataset is based on this dataset. We selected 813 out of 6113 genes using the same selection criteria as Iyer et al. [14] together with the criterion that the expression profiles had no missing data in the 24 arrays. Note that both datasets describe cyclic cell division processes. Specifically, the period of cell division in the human fibroblast cell-cycle is about 16 hours while the period of cell division in the yeast cdc15-synchronized cell cycle is 100 ± 10 minutes [13-16]. Table 1 summarizes these two datasets.

### Similarity Measures

Clustering algorithms for gene expression data need a similarity/distance measure. The choice of a similarity/distance measure may be as important as the choice of clustering algorithms. Two types of measures are extensively used in the comparison of gene expression profiles: correlation coefficient and Euclidean distance. For the following definitions assume that *g*_1 _= (*g*_11_, *g*_12_,⋯*g*_1*m*_) and *g*_2 _= (*g*_21_, *g*_22_,⋯*g*_2*m*_) represent the temporal expression profiles for genes *g*_1 _and *g*_2_, respectively, where *m *is the number of time points, and *g*_*ij *_represents the expression value of gene *i *at time point *j*.

*Correlation coefficient *is defined as:



where *g*_*ioffset *_(*i *= 1, 2) are two constants. The correlation coefficient *r*(·,·) has the range of [-1, 1]; specifically, *r*(*g*_1_, *g*_2_) = 1 means that genes *g*_1 _and *g*_2 _have a co-regulated response to a biological process in a same direction, and *r*(*g*_1_, *g*_2_) = -1 means that genes *g*_1 _and *g*_2 _have a co-regulated response to a biological process in an opposite direction. When *g*_*ioffset *_(*i *= 1, 2) are set to the means of expression profiles of genes *g*_1 _and *g*_2_, respectively, *r*(*g*_1_, *g*_2_) becomes the Pearson correlation coefficient of genes *g*_1 _and *g*_2_.

*Euclidean distance *is defined as:



The Euclidean distance measures the absolute distance between two genes in the expression space. It should be noted that if gene expression profiles are normalized such that their means are zero and their variances are one, the Euclidean distance is equivalent to the Pearson correlation measure because of the relationship *d*(*g*_1_, *g*_2_) = 2(1 - *r*(*g*_1_, *g*_2_)). In this case, the distance measure (*g*_1_, *g*_2_) = 1 - *r*(*g*_1_, *g*_2_) may be used [6].

### Hierarchical Clustering Techniques

**Figure 1:**
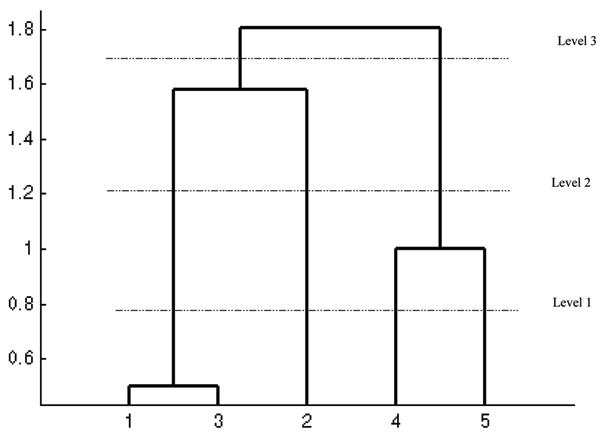
**A dendrogram of hierarchical clustering of 5 objects**. The numbers on the horizon axis represent the indices of objects, and the numbers on the vertical axis represent the distance between any two objects connected. In this example, objects 1 and 3 are merged in the first level; object 4 and 5 are merged in the second level; objects (1, 3) and 2 are merged in the third level; and finally objects (1, 2, 3) and (4, 5) merged in the fourth level. when the dendrogram is cut at the fourth level, two clusters (1, 2, 3) and (4, 5) are obtained; When the dendrogram is cut at the third level, three clusters (1, 2), (3) and (4, 5) are obtained; and so on.

**Figure 2:**
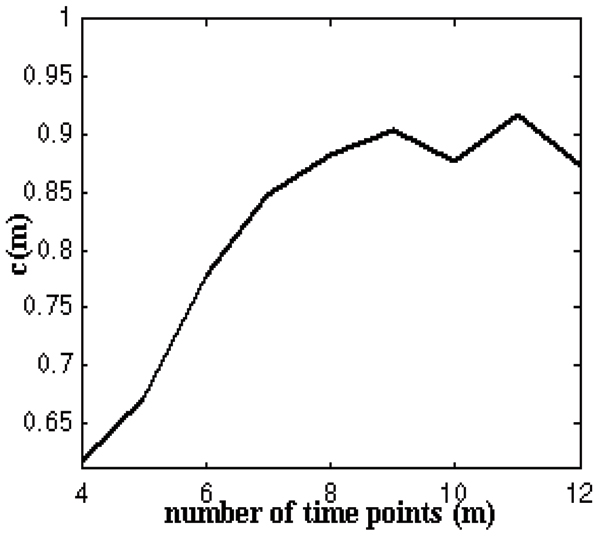
Profile of function *c(m) *with respect to the number of time points for the fibroblast experiment.

**Figure 3:**
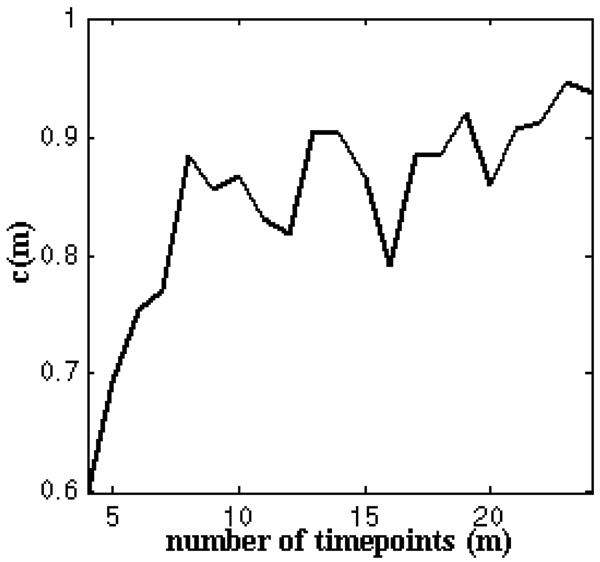
Profile of function *c(m) *with respect to the number of time points for the cdc15 experiment.

**Figure 4:**
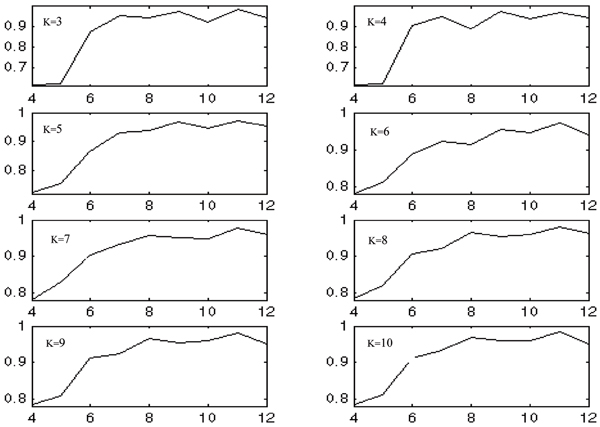
Profiles of function *d(k, m) *with respect to both the number of time points and the number of clusters for the fibroblast experiment (Iyer et al., 1999). The horizon axis presents the number of time points *m *while the vertical axis presents *d(·, m)*. From the upper-left corner to the lower-right corner, the 8 panels are for K = 3, 4,...,10, respectively.

Assume that there are *n *objects (e.g., genes). The task of clustering is to group the *n *objects into a number of subgroups based on a particular similarity measure and an algorithm of a particular clustering technique. Hierarchical clustering techniques are widely used for analyzing gene expression data [6,14,15]. There are basically two types of hierarchical clustering techniques in terms of their general procedures: agglomerative and divisive. An *agglomerativ*e procedure starts with *n *singleton clusters and successively merges clusters with the maximum similarity. The *divisive *procedure starts with all objects in one cluster and successively splits clusters such that the resultant clusters have the minimum inter-cluster similarity. The agglomerative procedure usually enjoys simpler computational complexity than the divisive procedure. In the literature, the agglomerative procedure is also called the bottom-up or clumping procedure, while the divisive procedure is called the top-down or splitting procedure. In this paper, the agglomerative technique of the hierarchical clustering is used. The result of a hierarchical clustering can be visualized by a binary tree called a dendrogram which shows how the clusters (or objects) are related to each other (see Figure 1). For *n *objects, there are *n *- 2 different levels that cut the tree. Each possible cut corresponds to a partition of the *n *objects. Therefore, the dendrogram of *n *objects may produce *n *- 2 different partitions with *k *= 2,⋯,*n *- 1 clusters. For instance, cutting at level 1 (see Figure 1) leads to the partition with four clusters: (1, 3), (2), (4), (5), while cutting at level 2 leads to the partition with three clusters: (1, 3), (2), (4, 5), and so on.

It is important to know how similar the clustering based on gene expression data at the first *m *time points is to the clustering based on data at the first *m *+ 1 time points. This issue has received considerable attention. A number of similarity measures for hierarchical clusterings have been proposed [17-19]. The general idea behind the similarity measure for two clusterings used here is that the difference between two clusterings can be evaluated by examining where a pair of objects lies in any two clusterings [17]. An object pair is called *clustering-invariant *if two objects of that pair stay either in the same clusters or in different clusters of two different clusterings. It is noted that the hierarchical clustering algorithm has a deterministic number of iterative steps. The first iteration corresponds to the first level of cut on the dendrogram (see Figure 1), and so forth. The hierarchical clustering of *n *objects has *n *- 1 iterations. Denote *C*(*i*, *j*) as an iteration index (*l*) at which objects *i *and *j *are merged into one cluster for the first time, i.e. *C*(*i*, *j*) = *l*. In the literature, *C *= [*C*(*i*, *j*)]_*n *× *n *_is called the index cophenetic matrix [18,19]. It is interesting to note that the index cophenetic matrix fully describes the topology of the dendrogram of a corresponding hierarchical clustering. With the index cophenetic matrix, Sokal and Rohlf [19] employed the gamma index proposed by Goodman and Krusal [20] to measure the similarity of their corresponding clusterings. The gamma index was shown to be one of the best similarity measures of hierarchical clusterings [17,18]. In the presented method, we employed this measure.

Given two clusterings, we have two index cophenetic matrices, *C*_1 _and *C*_2_. The gamma index is defined as the difference between two conditional probabilities for two object pairs selected at random from all possible object pairs and untied in two index cophenetic matrices *C*_1 _and *C*_2_, respectively; i.e.,

*γ*(*C*_1_, *C*_2_) = *p*(same ordering | untied pairs) - *p*(different ordering | untied pairs)     (3)

where *p*() stands for the probability that the events in the parentheses occur. The Gamma index has the range of [-1, 1] where the value 1 indicates a perfect agreement between two hierarchical clusterings. To avoid the negative probability, we adopt the following index:

*γ*_*c*_(*C*_1_, *C*_2_) = *p*(same ordering | untied pairs)     (4)

Further,



because *p*(same ordering | untied pairs) + *p*(different ordering | untied pairs) = 1. The index *γ*_*c *_has the range of [0, 1] where the value 1 indicates a perfect agreement between two hierarchical clusterings. Hays [21] provided a discussion on the computational aspects for index *γ*_*c*_.

Two hierarchical clusterings can also be compared with two particular partitions obtained from their dendrograms. To measure the similarity of the two partitions, we first define a new matrix, which is slightly different from the index cophenetic matrix, as follows:



In fact, the *D *matrix of a partition with *k *clusters can be obtained from matrix *C *by setting *D*(*i*, *j*) = 1 when *C*(*i*, *j*) ≤ *n *- *k*, and *D*(*i*, *j*) = 2 otherwise. Likewise an index denoted by *γ*_*D*_, similar to *γ*_*c*_, can be defined, which has the same form of an expression as Equation (5) except that *C*_1 _and *C*_2 _are replaced by *D*_1 _and *D*_2_. The higher *γ*_*D*_, the more similar are the two partitions.

### Algorithm, complexity and implementation

The basic idea of our method is to measure how similar a clustering produced from gene expression data from the first *m *time points is to a clustering produced from gene expression data from the first *m *- 1 time points. Denote the index cophenetic matrix corresponding to the first *m *time points by *C*_*m*_. Here the index *m *begins with 3 because the pattern discovery of gene expression from only 2 time points is trivial when data normalization methods [6] are applied. Further, define:

*c*(*m*) = *γ*_*c*_(*C*_*m *- 1_, *C*_*m*_), for any *m *≥ 3     (6)

where *C*_2 _is an arbitrary symmetrical matrix valued from the set {1,⋯,*n *- 1}, and *γ*_*c*_(*C*_*m *- 1_, *C*_*m*_) is calculated from Equation (5). *c*(*m*) for *m *≥ 3 is clearly a function of the number of time points, *m*. The larger *c*(*m*), the more similar are the two clusterings obtained from the first *m *time points and the first *m *- 1 time points. Therefore, the determination of the minimum number of microarray experiments corresponds to the determination of m* such that *c*(m*) is a maximum.

The rationale for the idea described above is as follows: We assume that given a set of gene expression data, the interesting patterns (clusters in this case) are inherently present. The characteristics of a particular pattern are described by the observed features (expression values) of genes involved in a biological process under investigation. The discriminant characteristics of the patterns are "bounded" such that there is a threshold beyond which any further observation will not add any value to the discrimination of patterns. In other words, such patterns can be eventually discovered in a limited number of experiments.

Similarly, denote by  the index cophenetic matrix of a partition with *k *clusters from a hierarchical clustering based on gene expression data from the first *m *time points. We can define a similarity function *d*(*k*, *m*) based on , i.e.



The computational complexity of the method above is analyzed as follows. For some given *m *and *k*, cophenetic matrices *C*_*m *_and  can be computed in time *O*(*n*^3^) by a hierarchical clustering algorithm such as that proposed by Duda et al. [22]. Both values *c*(*m*) and *d*(*k*, *m*) can be computed in time *O*(*n*^2^) [21]. Since the numbers of time points and the number of clusters are much less than the number of objects, *n*, the overall complexity is *O*(*n*^3^).

According to what similarity measure is used, the agglomerative hierarchical clustering techniques may further be classified into the *single linkage clustering*, *complete linkage clustering*, or *average linkage clustering *[22,23]. The average linkage technique of hierarchical clustering is used in this work.

The proposed method was implemented in MATLAB, using average linkage hierarchical clustering and Euclidean distances. The data were pre-processed by the method proposed by Eisen et al. [6]; specifically, we alternatively adjusted medians for genes and arrays six times, and then normalized each gene expression profile to have the variance of one before clustering.

## Authors' contributions

FXW proposed the idea of this paper, implemented the programs, and drafted the manuscripts. WJZ and AJK conceived of the study and helped to modify the manuscript. All authors read and approved the final manuscript.

## Figures and Tables

**Table 1:** The summary of two datasets.

Name of dataset	# of time points	# of selected genes	Period
fibroblast	12	517	About 16 hours
cdc15	24	813	100 ± 10 minutes

**Table 2:** *c*(*m*) for the fibroblast experiment. The bold number is the value at which *c*(*m*) reaches a maximum for the first time.

*m*	4	5	6	7	8	9	10	11	12
*c*(*m*)	0.6162	0.6715	0.7786	0.8486	0.8830	**0.9030**	0.8775	0.9184	0.8732

**Table 3:** *c*(*m*) for the cdc15 experiment. The bold number is the value at which *c*(*m*) reaches a maximum for the first time.

*m*	4	5	6	7	8	9	10	11	12	13
*c*(*m*)	0.6030	0.6940	0.7545	0.7713	**0.8847**	0.8562	0.8670	0.8305	0.8187	0.9046

*m*	14	15	16	17	18	19	20	21	22	23

*c*(*m*)	0.9023	0.8659	0.7913	0.8834	0.8835	0.9201	0.8600	0.9063	0.9120	0.9466
